# Employees' perception of digital human resource management changes and proactive behavior: the mediating role of work engagement and moderating effect of person-organization fit

**DOI:** 10.3389/fpsyg.2025.1623702

**Published:** 2025-09-26

**Authors:** Xiaogang Zhou, Qingguo Xiong, Miaoqiao Wang, Ling Huang, Mingyu Zhong

**Affiliations:** ^1^School of Economics and Management, East China Jiaotong University, Nanchang, China; ^2^Basic Teaching Department, Jiangxi College of Foreign Studies, Nanchang, China

**Keywords:** digital human resource management change perception, proactive change behavior, work engagement, person-organization fit, employee adaptation

## Abstract

To keep pace with the evolving needs of enterprise development, Human Resource Management (HRM) must embrace digital and intelligent transformation. However, organizational change is inherently risky and unpredictable, and employees' willingness to proactively engage in such changes remains uncertain. Drawing on Social Cognitive Theory (SCT), Self-Determination Theory (SDT), and the Ability-Motivation-Opportunity (AMO) model, this study explores how employees' perceptions of digital-intelligent HRM change influence their proactive change behavior. Work engagement is introduced as a key mediating mechanism in this relationship. Person-organization fit serves as a significant moderator between work engagement and proactive change behavior, ultimately leading to greater employee enthusiasm. First, based on 390 valid responses, the study reveals that employees' perception of digital-intelligent HRM change has a positive impact on proactive change behavior. Second, work engagement partially mediates this relationship. Third, person-organization fit negatively moderates the relationship between work engagement and proactive change behavior. These findings suggest that managers should recognize the critical role of employees during organizational change, create a supportive environment for change, communicate change-related information effectively, and establish open feedback channels to encourage employees at all levels to engage in the change process.

## 1 Introduction

Digital transformation is reshaping human resource management through platformized self-service, real-time analytics, and AI-supported decisions, changing how enterprises manage human capital and optimize internal operations. These technologies foster business-model innovation, thereby refining existing models. In this context, we focus on employees' perceptions of digital-intelligent HRM change as a central determinant of whether they engage with and proactively support organizational transformation (Do et al., 2025; Liu et al., 2025).

As organizations adopt digitalization and virtual structures, information management becomes more centralized (Zhang-Zhang et al., 2022). In a Volatile, Uncertain, Complex, And Ambiguous (VUCA) environment, management teams emphasize developing employee potential to enhance organizational agility (Shet, 2024). Employees increasingly participate in decision-making, and their willingness to initiate change is critical for transformation (Qiao et al., 2024). The organization—employee relationship has shifted from a transactional arrangement toward a more collaborative partnership (Yalenios and d'Armagnac, 2023). To leverage employee agency and creativity, HRM grounded in openness and connectivity should integrate networked information technologies. Doing so elevates employees' roles and makes their accurate understanding of digital transformation objectives pivotal to the success of digital-intelligent HRM change.

Organizational change is complex and context-dependent, shaped by employee attitudes and managerial practices (Fu et al., 2023). Prior research shows that change strategies can improve performance and support sustainable development. Empirical work links change to better organizational outcomes (Tripathi and Kalia, 2024). Proactive change behavior refers to voluntary, persistent efforts by employees to drive functional changes in jobs, departments, or organizations (Ma et al., 2023). Such behavior is challenging, innovation-oriented, and constructive, often requiring extra time and effort beyond routine duties (Batool et al., 2023). Some employees, however, prefer to maintain the status quo and focus on assigned tasks, which can impede effective change implementation. Employees' perceptions of change, encompassing the identification, appraisal, and assimilation of change-related information, strongly influence psychological states and job outcomes. They shape happiness and turnover intentions via work engagement and burnout (Apaydin et al., 2023; Buttigieg et al., 2023; Lim and Moon, 2024) and can weaken the link between desire for control and voice (Lu and Lu, 2020).

Despite these insights, important gaps remain. Much scholarship emphasizes the negative consequences of change, such as reduced job satisfaction and increased stress (Backhaus et al., 2024; Cao et al., 2024), and comparatively underexamines employees' perceptions of change as a driver of proactive behavior. Existing work that touches this link is limited. For example, qualitative evidence suggests that perceived importance and self-efficacy motivate taking charge (Ngo et al., 2023). What is underexplored is how these relationships operate within digital-intelligent HRM change, where algorithmic processes heighten change cues, may support or strain autonomy, competence, and relatedness, and reconfigure ability, motivation, and opportunity conditions. Consequently, it remains unclear when engagement is more or less likely to translate into proactive change under this data-rich HRM setting. This gap provides the motivation for the present study.

This study examines how employees' perceptions of digital-intelligent HRM change relate to proactive change behavior, with work engagement as a mediator and person-organization fit as a moderator. Drawing on SCT, SDT, and the AMO framework, we theorize context-specific micro-foundations: heightened and continuous change cues in line with SCT, potential tensions and supports in autonomy, competence, and relatedness consistent with SDT, and redesigned ability, motivation, and opportunity structures associated with AMO that together shape whether perceptions of change channel engagement into taking charge. Importantly, we argue and test a counter-intuitive boundary condition: higher person-organization fit attenuates the engagement → proactive change linkage during digital-intelligent HRM change, which leaves less marginal scope for additional engagement to produce incremental change-oriented action.

This research makes three contributions. First, it embeds the perception → engagement → proactivity pathway in the digital-intelligent HRM context, articulating micro-foundations that differentiate this setting from conventional HRM, including heightened cues, variation in need support and strain, and redesigned AMO conditions. Second, it clarifies the mediating role of work engagement in channeling perceptions of digital-intelligent HRM change into proactive change behavior. Third, it identifies a theoretically grounded boundary condition by demonstrating that person-organization fit weakens the engagement-proactivity association in this context, thereby specifying when engagement yields smaller behavioral payoffs and advancing theory on change, engagement, and taking charge.

## 2 Research hypothesis

### 2.1 Change perception and employees' proactive change behavior

In the digital age, digital transformation has become essential for enterprises. By leveraging digital and intelligent information technologies, organizations can enhance HRM services for both employees and the organization, thereby facilitating the development and optimization of human resources. In the context of digital-intelligent HRM change, platformized self-service, real-time analytics, and AI-supported decisions make change cues more salient and frequent and reconfigure day-to-day workflows, which raises the behavioral stakes of how employees perceive change. From an SCT perspective, clear task cues and credible performance feedback strengthen employees' efficacy beliefs and outcome expectations, which energize self-regulated, change-oriented actions such as process improvement and problem solving (Lent et al., 2022). Proactive change behavior aims to improve organizational efficiency by optimizing work processes. Relative to traditional HR adjustments, digital-intelligent HRM change can also introduce technology load and AI-enabled monitoring, so employees' interpretations of what the change implies for their roles become especially consequential for whether they choose to take charge.

Employees' perception of change reflects their comprehension of the significance and necessity of organizational change. This includes aspects such as impact, intensity, importance, outcomes, and employees' perceived control over the change process (Lau and Woodman, 1995). Since organizational change often introduces uncertainty, it can evoke feelings of fear and loss among employees (Edwards et al., 2024). Specifically, a lack of clear understanding of the changes can lead to negative emotions that hinder the progress of the change (Khaw et al., 2023). However, organizational change is a two-way process. When reforms are appropriately implemented, they can inspire employees to take self-initiated actions to embrace the change (Gilstrap and Hart, 2020). As HRM continues to evolve within organizations, employee management models are also gradually transforming. Accordingly, when employees clearly understand the purpose, scope, and implications of a given change, they are more likely to align with organizational goals and adopt constructive, change-supportive behaviors that enhance performance (Brown, 2017).

Digital-intelligent HRM transformations enhance information-sharing services, enabling employees to access accurate data for decision-making and engage more effectively in organizational decisions. Beyond cognition, SDT suggests that when change practices support autonomy, competence, and relatedness through transparent workflows, usable tools, and developmental support, employees' high-quality motivation is strengthened, which in turn fosters proactive taking-charge behaviors (Almustafa et al., 2025). According to cognitive theory, individuals interpret the same situation differently based on their personal perspectives and characteristics, which results in varying perceptions of and responses to change (Chen et al., 2023). Given employees' differing interests in digital-intelligent HRM, their perceptions and behaviors toward change may vary widely. The AMO theoretical model posits that employees' proactive and constructive change behaviors are collectively influenced by three key factors: ability, motivation, and opportunity. Under digital-intelligent HRM change, practices that build skills, energize motivation, and expand participation create conditions in which engaged employees can more readily translate their perceptions into visible taking-charge behaviors (Harrell-Cook et al., 2001). Conversely, when employees perceive fewer opportunities or inadequate support to apply newly acquired abilities, the same level of engagement is less likely to convert into proactive improvements. To ensure that employees align their actions proactively with organizational change, managers should provide guidance that helps them develop a sense of identification with the change. Therefore, we propose the following hypothesis.

**Hypothesis 1:** Employees' perception of digital-intelligent HRM change positively influences their proactive change behavior.

### 2.2 Change perception and work engagement

The behavior of individuals is shaped by both their external environment and internal cognitive states, thus highlighting the significant interplay between cognitive activity and behavior. From an SCT perspective, clear and credible change cues strengthen self-efficacy and activate self-regulatory processes, such as forethought, self-monitoring, and self-reactive influence, thereby sustaining task effort and attentional focus that are central to engagement (Chen and Lin, 2024). Complementing this view, the AMO framework holds that work engagement is more likely when digital-intelligent HRM change enhance employees' ability, motivation, and opportunity, clarifying how practices that build skills, energize motivation, and expand participation create conditions for sustained engagement (Bos-Nehles et al., 2023). According to Roekel et al. (2023), work engagement encompasses three key components: vigor, dedication, and absorption. These components reflect a positive state of mind toward work that motivates employees to actively engage in their tasks (Gupta et al., 2024).

Employees typically seek to have their needs met, work in roles aligned with their abilities, and experience a sense of belonging within the organization. Research has shown that intrinsic motivation increases significantly when organizational change better meets employees' diverse needs (Nie et al., 2023). Al'Ararah et al. (2024) argue that organizational change is likely to result in shifts in various organizational practices and the regulations governing employees' work. These changes, in turn, affect the environment and the content of employees' daily tasks. As a result, employees may encounter new job structures, expectations, roles, and responsibilities (Bellou, 2008). This situation can trigger varying levels of psychological stress, primarily stemming from the uncertainty employees feel regarding change and their concern about potential negative outcomes. Such feelings of uncertainty can significantly reduce work engagement and inhibit proactive change behavior (Sun et al., 2022).

During organizational change, employees' work engagement is significantly influenced by their perception of the change. Studies have shown that employees' cognition and beliefs about organizational change can greatly enhance their sense of competence at work (Ling et al., 2024). According to SDT, when organizations implement credible changes in human resource policies to meet employees' needs and increase developmental opportunities, such changes strengthen employees' sense of belonging. This heightened sense of belonging enhances job satisfaction and fulfills employees' needs for learning, growth, and development, thereby improving work engagement (Battaglio et al., 2022). Conversely, when employees hold negative attitudes toward change, their motivation to work may decrease, resulting in behaviors such as low work engagement or resistance. Based on these insights, we propose the following hypothesis.

**Hypothesis 2:** Employees' perception of digital-intelligent HRM change positively influences their work engagement.

### 2.3 Work engagement and proactive change behavior

Cognition is a distinct psychological process that includes three key stages: obtaining information, storing it, and processing it. This process begins with the collection of information and culminates in the processing of that information, ultimately shaping an individual's cognition of a subject. Building on SCT, behavior is formed through the reciprocal interaction of the person, environment, and behavior. When the work context affords satisfaction and mastery cues, employees adjust their actions accordingly, reinforcing this triadic dynamic (Kytle and Bandura, 1978). Work engagement reflects the mobilization of employee motivation across physical, emotional, and cognitive dimensions (Li et al., 2024). When employees invest more time and energy in their work, it can have a positive impact on their wellbeing and proactive behavior. A substantial body of research indicates that work engagement positively affects employee job satisfaction, performance, and citizenship behaviors (Gürbüz et al., 2023), and reduces employees' turnover propensity (Abukhalifa et al., 2023). Additionally, work engagement effectively moderates the relationship between autonomy and performance, while also fostering innovative behavior among employees (Jindal et al., 2022; Tisu et al., 2023; Liu et al., 2024).

From a cognitive perspective, work engagement helps employees derive satisfaction from demanding tasks, thereby fostering the development of a positive mindset. Moreover, employees' level of work engagement is positively associated with their initiative (Xiao, 2024). As a positive self-regulatory state, work engagement reflects employees' efforts to maintain consistent performance (Liu et al., 2023). Consistent with SDT, when organizational practices support autonomy, competence, and relatedness, self-determined motivation increases and is more likely to translate into proactive behavior (Chua and Ayoko, 2021). In parallel, the AMO perspective explains how engagement channels resources into constructive action: when employees possess relevant skills, feel motivated to invest effort, and have opportunities to participate, they are more inclined to initiate and implement improvements (Liehr and Hauff, 2025). When enterprises implement digital-intelligent HRM practices, employees with higher work engagement are more likely to adapt to new work modes introduced by these changes and actively engage in reform efforts. Additionally, a high level of work engagement signifies greater focus, increased effort, and a higher ability to persevere. It also shows that engaged employees are more willing to take on proactive change behavior, even when those behaviors involve high risks and challenges. Accordingly, we propose the following hypothesis.

**Hypothesis 3:** Employees' work engagement positively influences their proactive change behavior.

### 2.4 The mediating role of work engagement

SDT posits that motivation and wellbeing depend on the satisfaction of three basic psychological needs: autonomy, competence, and relatedness. In organizational settings, employees typically require contextual support to satisfy these needs (Chi et al., 2020). When these needs are fulfilled, employees internalize extrinsic motives and sustain intrinsic motivation, which improves performance and yields more durable positive behavioral outcomes (Deci et al., 2017). Within this framework, work engagement functions as an energized, persistent state through which need-supportive contexts translate into action. Employees who are engaged invest additional time and effort and show sustained concentration on their tasks (Gerbeth and Mulder, 2023). Work engagement has been shown to significantly enhance employees' performance (Albrecht et al., 2023) and help employees cope with the demands of stressful jobs (Ni et al., 2024).

From a social cognitive perspective, clear change cues strengthen efficacy and self-regulation, sustaining the focused effort typical of engagement and helping convert change perceptions into action (Schunk and DiBenedetto, 2020). In parallel, the AMO view positions engagement as the motivational conduit through which HRM practices influence behavior, because ability- and opportunity-enhancing practices exert their effects largely via employees' motivation (Zahoor et al., 2024). Employees with higher work engagement are also more likely to grasp the purpose and significance of digital-intelligent HRM change. This understanding promotes positive adaptation to change and drives proactive change behavior. Conversely, when work engagement is low, reduced energy and focus are likely to undermine positive responses to this change.

**Hypothesis 4:** Work engagement mediates the relationship between employees' perception of digital-intelligent HRM change and their proactive change behavior.

### 2.5 The moderating role of person-organization fit

Person-organization fit refers to compatibility between individuals and organizations, which gradually develops through interaction and adaptation. This concept encompasses consistency matching, demand-ability matching, and demand-supply matching. Kristof-Brown et al. (2023) argue that the degree of alignment between individuals and organizations is an important aspect of organizational behavior. Specifically, it refers to the consistency of attributes, values, and culture between employees and the organization.

Through their interactions with the organization, employees develop a harmonious relationship, which helps reduce conflicts and misalignments. This alignment has positive effects on both individuals and the organization. Person-organization fit is closely related to employees' work behaviors, perceptions, and attitudes, significantly influencing their overall performance (Zeng and Hu, 2024). Studies have shown that the degree of matching can predict employee performance and is also indirectly influenced by organizational commitment (Goetz and Wald, 2022). Chatman (1989) found that as employees' tenure increases, their values, needs, abilities, and other personal characteristics gradually align with those of the organization, leading to a higher degree of matching. Additionally, studies have found that the relationship between employees and their organizations is positively correlated with employee satisfaction and career satisfaction, while being negatively correlated with employee turnover intentions (Lin and Huang, 2020; Tkalac Verčič and Men, 2023).

The alignment between individuals and organizations can be enhanced in several ways. One approach is by matching employees' skills with job requirements. Another way is to align employees' needs with job characteristics. Additionally, aligning employees' values with organizational values further strengthens the connection. These alignments foster greater employee identification with the organization and enhance their emotional attachment. When the match between an employee's abilities and job content satisfies both their personal and professional needs, it leads to higher work engagement (Vleugels et al., 2023).

A high degree of person-organization fit signals that the organization recognizes and values employees' qualities, sending signals that elicit positive emotions (Pan et al., 2020). In such cases, employees, regardless of their level of engagement, can meet job requirements without needing significant adjustments to their work methods. In contrast, when the alignment between the employee and the organization is low, it indicates that the employee's work does not meet organizational expectations. In this scenario, employees with high work engagement are more likely to take the initiative to address the situation, while those with low work engagement are less likely to make changes. From a SCT perspective, person-organization fit shapes discrepancy cues and outcome expectancies: under high fit, clearer norms and routinized, digitally standardized workflows reduce ambiguity and the perceived need for exploratory adjustments. Under low fit, misalignments are salient, which engaged employees attempt to close through agentic, change-oriented efforts. Consistent with SDT, high fit typically indicates that autonomy, competence, and relatedness needs are already satisfied, which can foster psychological comfort and reduce felt urgency. The marginal behavioral return to additional engagement is therefore lower. By contrast, low fit is accompanied by unmet needs, strengthening the motivation to invest effort in change to restore alignment. Viewed through the AMO lens, fit operates as a contextual opportunity structure: when fit is high, established routines already enable goal attainment, so creating additional opportunities via taking charge is less necessary. When fit is low, gaps in routines and processes create room for engagement to convert ability and motivation into proactive change. In digital-intelligent HRM settings, characterized by frequent, salient change cues, data transparency, and AI-supported decisions, these dynamics are amplified: high fit may encourage routinization and complacency, whereas low fit makes discrepancies more visible and sustains the impetus to act. Accordingly, we propose the following hypothesis.

**Hypothesis 5:** Person-organization fit moderates the relationship between employees' work engagement and proactive change behavior. Specifically, higher levels of fit weaken the positive effect of work engagement on proactive change behavior, whereas lower levels of fit strengthen this effect.

**Figure 1:**
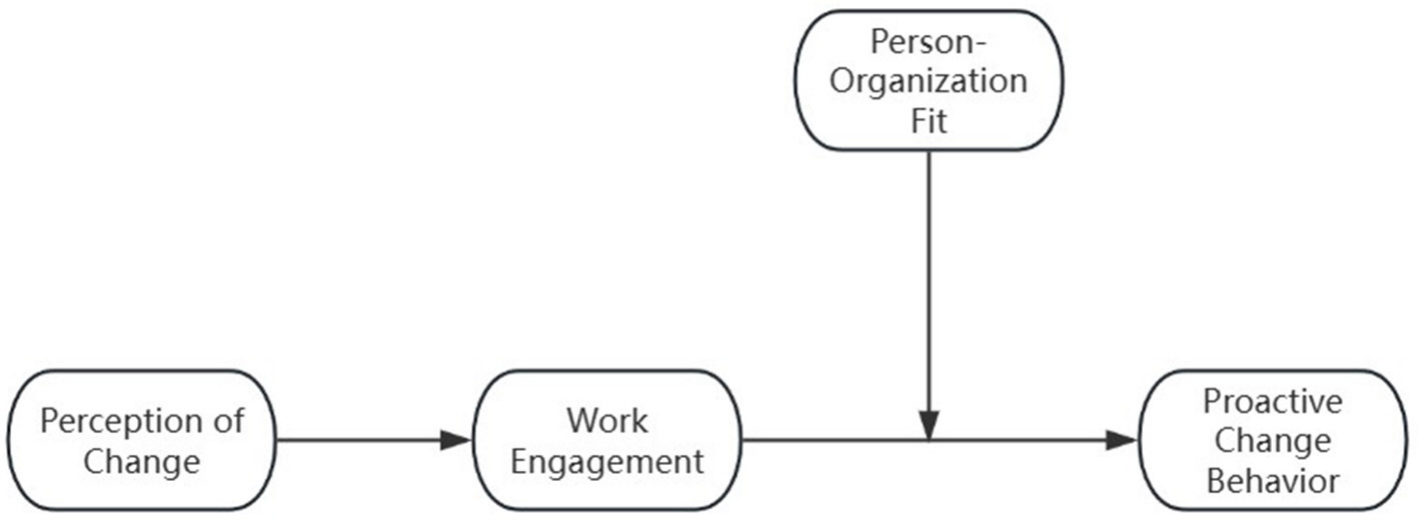
Research model framework.

Combining research variables and research hypotheses, the theoretical framework model for this study is constructed as shown in Figure 1.

## 3 Research design

### 3.1. Sample and data

The questionnaire for this study was primarily distributed to knowledge workers in various enterprises, and data were collected through an online survey. With the assistance of employed students, we contacted five large enterprises, each with more than 600 employees, located in Beijing, Hangzhou, Shenzhen, Nanchang, and other regions. These enterprises included private companies, state-owned enterprises, joint ventures, and foreign-funded enterprises, ensuring diverse representation across organizational levels. Stratified sampling was employed to gather samples from industries such as manufacturing, real estate, finance, the internet, and services, which ensured comprehensive coverage of multiple sectors. This approach minimized variability across sampling layers and enhanced the representativeness of the sample data. A total of 421 completed questionnaires were collected over a period of 3 weeks. Since all questions in the survey were mandatory, there were no invalid responses resulting from incomplete questionnaires. To ensure the data's validity, responses were screened according to methods used in previous research. Invalid samples, such as those with identical answers across all items, excessive or insufficient response time, or unreasonable basic information, were excluded. As a result, 31 invalid questionnaires were discarded, leaving 390 valid responses and yielding an effective response rate of 92.6%.

In terms of demographic distribution, gender was fairly balanced, with 233 females (59.7%) and 157 males (40.3%). Respondents' ages were mainly distributed across three groups: 26-30 years (85 people), 31-35 years (140 people), and 36-40 years (89 people), accounting for 80.5% of the sample. Regarding education, the majority of respondents had completed undergraduate or junior college education, with 183 and 169 respondents, respectively, accounting for 46.9% and 43.3% of the sample. Most participants (66.2%) had been with their organizations for over 3 years. Additionally, 66.2% of the participants were non-managerial employees, aligning with the study's objective of capturing grassroots employees' perspectives. Regarding the nature of the enterprises, 54.9% (214 respondents) worked in private enterprises. Similarly, 58 (14.9%) were employed in state-owned enterprises, 69 (17.7%) in joint ventures, and 49 (12.6%) in foreign-funded enterprises. Finally, regarding industry distribution, 17.7% (69 respondents) came from manufacturing, 14.9% (58 respondents) from internet-related sectors, 24.9% (97 respondents) from the services sector, and 18.7% (73 respondents) from real estate. Fewer respondents were from finance (9.7%, or 38 respondents) and other industries (3.1%, or 12 respondents).

### 3.2. Measuring scale

The scales employed in this study are well-established instruments widely utilized by both domestic and international scholars. Because the original instruments were developed in English, this study implemented a two-step translation procedure to ensure linguistic accuracy and cultural relevance: First, one bilingual expert produced a Mandarin translation. Second, an independent bilingual expert conducted a back-translation into English. Discrepancies between the source and back-translated versions were reviewed and reconciled by this study to preserve conceptual equivalence. Minor wording adjustments were made to align items with commonly used HR terminology in Chinese organizations. No items were added or deleted, and no changes were made to the constructs' factor structures. A 5-point Likert scale was used to assess the degree of agreement, with 1 indicating “strongly disagree” and 5 indicating “strongly agree” for each item. Unless otherwise noted below, scale scores were computed as the mean of their constituent items. Evidence of reliability and validity for the translated measures is reported in the Results (EFA/CFA) section.

Perception of Change: The transformational measurement scale developed by Reddick (2009) was used. Six items were retained based on conceptual relevance to digital-intelligent HRM change and content coverage. Responses were recorded on a 5-point Likert scale (1 = strongly disagree, 5 = strongly agree). A typical item is: “I believe the integration of HRM and information technology can make human resource managers more effective.”

Proactive Change Behavior: The scale measuring employee proactive change behavior, adapted by Fuller et al. (2012) from the scale of Morrison and Phelps (1999), was used. The instrument comprises six items. Responses were recorded on a 5-point Likert scale (1 = strongly disagree, 5 = strongly agree), and the mean item score was used as the composite. A typical item is: “I often try to introduce new structures, techniques, or methods to improve efficiency.”

Work Engagement: The Utrecht Work Engagement Scale (UWES), a 9-item scale developed by Schaufeli et al. (2006), was used to measure work engagement. The scale evaluates work engagement across three dimensions: vigor, dedication, and absorption. In the primary analyses, we used the global UWES-9 mean score, consistent with prior research. The three sub dimensions are defined as vigor (energy and persistence), dedication (involvement and significance), and absorption (concentration). A typical item is: “In my work, I feel strong and energetic.”

Person-Organization Fit: The scale developed by Cable and DeRue (2002) was used to assess person-organization fit, comprising 9 items across three dimensions. Items were averaged to form the overall fit score. A typical item is: “My values are very similar to the values advocated by the organization's value system.”

Additionally, to control for the potential influence of personal factors and organizational characteristics on the research outcomes, the study included gender, age, educational background, years of work experience, position, the nature of the organization, and industry type as control variables. These controls follow conventions in related HRM and organizational behavior research.

### 3.3 Ethics statement

This study was conducted in accordance with the Declaration of Helsinki, and the protocol was approved by the Ethics Committee (HREC) of the School of Economics and Management at East China Jiaotong University. The participants provided their written informed consent to participate in this study.

## 4 Data analysis

### 4.1. Reliability and validity test

Using the data from 390 responses, the reliability and validity of the scales were assessed through exploratory factor analysis conducted with SPSS software. The results are presented in Table 1. The Cronbach's α coefficients for each variable in this study were 0.904, 0.899, 0.941, and 0.932, respectively, all of which exceed the commonly accepted threshold of 0.85. These results indicate that the scale demonstrates good internal consistency and reliability. The Kaiser-Meyer-Olkin measure and Bartlett's Test of Sphericity further support the validity of the scale. The KMO values for all variables were greater than 0.9, and the overall KMO value for the scale was 0.948. Additionally, Bartlett's test yielded a significance coefficient less than 0.05, confirming that the scale variables are strongly correlated and justifying the use of factor analysis for further investigation.

**Table 1:** Validity analysis result.

**Variable**	**Variable Cronbach's α**	**KMO-value**	**Bartlett's-value**	**Item**
Perception of change	0.904	0.916	0.000	6
Proactive change behavior	0.899	0.912	0.000	6
Work engagement	0.941	0.958	0.000	9
Person-organization fit	0.932	0.959	0.000	9

### 4.2. Confirmatory factor analysis

To test the discriminant validity of each variable in the model, this study conducted a confirmatory factor analysis (CFA) on the questionnaire data. The model tested included four factors: perception of change, work engagement, proactive change behavior, and person-organization fit. As shown in Table 2, the four-factor model demonstrated the best fit (χ^2^ = 470.504, *df* = 399, $\frac{\chi^{2}}{df}\;=\;1.179$, RMSEA = 0.021, NFI = 0.941, CFI = 0.99). All parameters of the four-factor model met the standard requirements, and the model provided the best fit compared to the alternative models. Additionally, the Composite Reliability (CR) was greater than 0.9, and the Average Variance Extracted (AVE) value exceeded 0.6. These results indicate that the four-factor model has good discriminant validity.

**Table 2:** Confirmatory factor analysis.

**Model**	**χ^2^**	**df**	**χ^2^/df <3**	**RMSEA <0.08**	**NFI >0.9**	**CFI >0.9**
Four-factor model: WE, POF, PCB, PC	470.504	399	1.179	0.021	0.941	0.990
Three-factor model: WE+POF, PCB, PC	2398.234	402	5.943	0.113	0.700	0.736
Two-factor model: WE+POF+PCB, PC	3439.018	404	8.512	0.139	0.568	0.597
Single-factor model: WE+POF+PCB+PC	4338.960	405	10.713	0.158	0.455	0.477

PC, Perception of Change; WE, Work Engagement; PCB, Proactive Change Behavior; POF, Person-organization fit.

### 4.3. Correlation analysis

Descriptive statistical indicators, including the mean, standard deviation, and correlation coefficients for all variables, were calculated using SPSS software. Pearson correlation analysis was conducted to examine the relationships among change perception, work engagement, person-organization fit, and proactive change behavior in the context of digital-intelligent HRM. As shown in Table 3, perception of digital-intelligent HRM change was significantly positively correlated with employees' proactive change behavior (*r* = 0.36, *p* < 0.01) and with work engagement (*r* = 0.34, *p* < 0.01). These preliminary findings support Hypotheses 1 and 2. Additionally, work engagement is positively correlated with proactive change behavior (*r* = 0.31, *p* < 0.01). This provides preliminary evidence for Hypothesis 3, which posits that work engagement influences employees' proactive change behavior.

**Table 3:** Correlation analysis table (*N* = 390).

**Values**	***M***	***SD***	**1**	**2**	**3**	**4**	**5**	**6**	**7**	**8**	**9**	**10**
1. Gender	1.59	0.49										
2. Age	2.96	1.11	−0.12^*^									
3. Educational background	1.69	0.72	−0.06	0.10								
4. Year of experience	3.24	1.17	0.02	0.59^**^	0.08							
5. Position	1.49	0.79	−0.04	0.23^**^	0.05	0.21^**^						
6. Industry	2.28	0.87	−0.09	0.14^**^	0.01	0.08	0.03					
7. Type of enterprise	3.32	1.66	−0.03	0.03	0.00	−0.01	0.09	0.02				
8. Perception of change	3.81	0.83	−0.01	0.06	0.05	0.05	0.04	−0.02	0.02			
9. Proactive change behavior	3.78	0.83	0.03	0.05	0.07	−0.03	0.02	0.01	0.08	0.36^**^		
10. Work engagement	3.77	0.87	0.01	0.12^*^	0.06	0.06	0.08	−0.04	0.00	0.34^**^	0.31^**^	
11. Person-organization fit	3.82	0.82	0.07	0.01	0.08	0.03	−0.05	−0.12^*^	−0.01	0.47^**^	0.29^**^	0.34^**^

M, Mean; SD, Standard Deviation; ^*^p < 0.05; ^**^p < 0.01.

## 5 Hypothesis test result

### 5.1. Model evaluation

In this study, AMOS software was used to analyze the relationships among perception of change, proactive change behavior, and work engagement. As shown in Table 4, the main fit indices, CFI = 0.986, IFI = 0.986, and TLI = 0.985, are all above the recommended threshold of 0.90. Additionally, CMIN/DF is 1.377 (less than 3), and RMSEA is 0.038 (less than 0.05), indicating a good model fit. These results indicate good overall model fit.

**Table 4:** Model fitting index values.

**Indicators**	**Evaluation criterion**	**Model results**	**Fitting**
CMIN/DF	≤3.0	1.377	Ideal
RMSEA	≤0.08	0.038	Ideal
IFI	≥0.90	0.986	Ideal
TLI	≥0.90	0.985	Ideal
CFI	≥0.90	0.986	Ideal
NFI	≥0.90	0.952	Ideal
RFI	≥0.90	0.946	Ideal
GFI	≥0.90	0.941	Ideal
PNFI	≥0.50	0.844	Ideal
PCFI	≥0.50	0.874	Ideal

### 5.2. Path analysis

**Figure 2:**
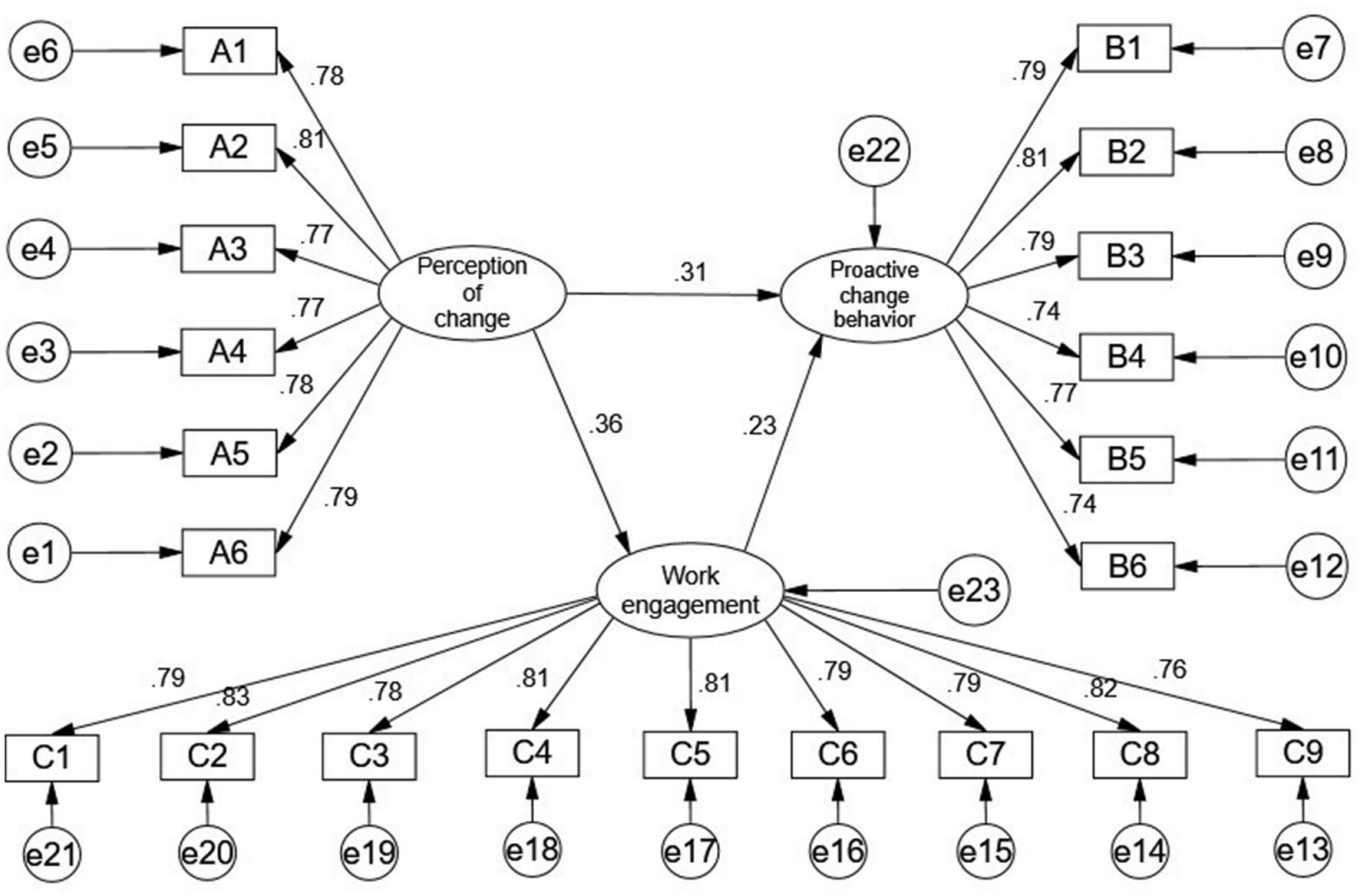
Standardized path coefficient diagram.

In this study, AMOS software was used to analyze the relationships among perception of change, work engagement, and proactive change behavior. The standardized path coefficients are presented in Figure 2. According to the path coefficient test results shown in Table 5, the following conclusions can be drawn: First, the perception of digital-intelligent HRM change was significantly positively correlated with proactive change behavior (standardized path coefficient = 0.306, *p* < 0.001). This indicates that when employees positively perceive digital-intelligent HRM change, they tend to adopt a more positive attitude toward organizational reform, making them more likely to understand and accept the relevant aspects of the change. In other words, a higher degree of positive perception facilitates the emergence of proactive change behavior. Therefore, Hypothesis 1 is supported. Second, the perception of digital-intelligent HRM was significantly positively correlated with work engagement (standardized path coefficient = 0.365, *p* < 0.001), indicating that Hypothesis 2 is supported. When employees perceive change positively, they are more likely to recognize its benefits and thus become more engaged in their work. Third, employee work engagement was significantly positively correlated with proactive change behavior (standardized path coefficient = 0.227, *p* < 0.001), supporting Hypothesis 3. This suggests that employees with higher levels of work engagement are more focused, better able to understand and support organizational changes, and more likely to exhibit proactive change behavior. Specifically, when employees are more engaged, they tend to have a stronger sense of organizational belonging and are more willing to undertake positive change behaviors that align with organizational development.

**Table 5:** Model parameter estimation.

**Variable**	**Standardized path coefficient**	**S.E**.	**C.R**.	***p*-value**
Work engagement ← Perception of change	0.365	0.060	6.521	0.000
Proactive change behavior ← Work engagement	0.227	0.056	4.038	0.000
Proactive change behavior ← Perception of change	0.306	0.062	5.246	0.000

### 5.3. Mediation effect testing

This study used AMOS software and the Bootstrap method to examine the mediating effect of work engagement on the relationship between change perception and employees' proactive change behavior. The test was performed with 5,000 resamples and a 95% confidence interval. The detailed Bootstrap results are presented in Table 6. Compared with the path coefficient results in Table 5, the Bootstrap results are consistent in terms of magnitude, significance, and direction of the regression coefficients. From the perspective of total effect analysis, the total effect of change perception on proactive change behavior is 0.414. At the 95% confidence level, the bias-corrected percentile confidence interval is (0.264, 0.585), which excludes zero, and the *Z*-value is 5.111 (>1.96), indicating a significant positive total effect. Regarding the indirect effect, the effect of change perception on proactive change behavior through work engagement is 0.088. The 95% confidence interval is (0.038, 0.174), which excludes zero, and the *Z*-value is 2.667 (>1.96). This result confirms that work engagement significantly mediates the relationship between change perception and proactive change behavior. As for the direct effect, the direct effect of change perception on proactive change behavior is 0.326, with a 95% confidence interval of (0.171, 0.510), which also excludes zero. The corresponding *Z*-value is 3.747 (>1.96), indicating that the direct effect is statistically significant. In summary, Hypothesis 4 is supported. Work engagement serves as a partial mediator between employees' perception of digital-intelligent HRM change and their proactive change behavior.

**Table 6:** Bootstrapping mediation effects testing.

**Summary of the hypothesized path**	**Point estimate**	**Product of coefficients**		**Bootstrap 5000 times 95% CI**			
				**Bias corrected**		**Percentile**	
		**S.E**.	**Z**	**LL**	**UL**	**LL**	**UL**
Indirect effects	0.088	0.033	2.667	0.038	0.174	0.033	0.162
Direct effects	0.326	0.087	3.747	0.171	0.51	0.168	0.508
Total effect	0.414	0.081	5.111	0.264	0.585	0.264	0.587

LL, Lower limit; UL, Upper limit.

### 5.4 The moderating role of person-organization fit

This study employed hierarchical regression and the Bootstrap method to examine the moderating effect of person-organization fit on the relationship between employee's work engagement and proactive change behavior. Three models were constructed for data analysis. In Model 1, employee proactive change behavior was the dependent variable, and control variables were included in the regression model. Model 2 used work engagement as the independent variable and person-organization fit as the moderating variable to assess their impact on employee's proactive change behavior. In Model 3, the interaction term between work engagement and person-organization fit was added to the regression model. A significant interaction term would indicate the presence of a moderating effect. To address multicollinearity, work engagement and person-organization fit were centered before forming the interaction term. The regression analysis results of work engagement, person-organization fit, and their interaction are presented in Table 7. In addition, we used PROCESS to probe conditional effects with the Johnson-Neyman (J-N) technique based on the mean-centered moderator. We also computed simple slopes at low (−1 SD) and high (+1 SD) levels of person-organization fit while holding covariates at their means to facilitate interpretation.

**Table 7:** Analysis of the moderating effects of person-organization fit.

**Type of variable**	**Variable name**	**Model 1**	**Model 2**	**Model 3**
Control variable	Gender	0.055	0.035	0.040
	Age	0.094	0.059	0.048
	Educational background	0.073	0.044	0.036
	Year of experience	−0.088	−0.087	−0.084
	Position	0.004	0.002	0.000
	Industry	0.005	0.042	0.033
	Type of enterprise	0.078	0.080	0.076
Independent variable	Work engagement		0.239^***^	0.211^***^
Moderating variable	Person-organization fit		0.210^***^	0.180^***^
Interaction term	Work engagement^*^ Person-organization fit			−0.187^***^
Model summary	*R* ^2^	0.020	0.151	0.188
	Adjusted *R*^2^	0.002	0.131	0.166
	*F*	1.114	7.536	8.765

^*^Indicates significant at 0.05 level; ^**^indicates significant at 0.01 level; ^***^means significant on the 0.001 level.

The regression analysis results indicate that the *R*^2^ of Model 3, which includes interaction terms, is higher than that of Models 1 and 2, which lack interaction terms. Additionally, the regression coefficient of the interaction term between work engagement and person-organization fit was significant (*p* < 0.001), suggesting that person-organization fit significantly affects the relationship between work engagement and proactive change behavior. Specifically, the regression coefficient for this interaction term was −0.187 (*p* < 0.001), indicating that person-organization fit negatively moderates the relationship between work engagement and proactive change behavior. Notably, the main effects in Model 3 remain positive for work engagement (β = 0.211, *p* < 0.001) and for person-organization fit (β = 0.180, *p* < 0.001), while their combination attenuates the marginal effect of engagement as fit increases. This pattern implies that engagement is behaviorally most consequential under lower fit and least consequential under higher fit. The J-N analysis from PROCESS identified a single threshold on the mean-centered fit scale at 0.433. When person—organization fit is at or below 0.433, the simple effect of work engagement on proactive change behavior is significantly positive at the 95% level, whereas above this value the effect is not significant. To test whether this pattern holds across the UWES-9 facets, we re-estimated PROCESS (Model 14) three times using vigor, dedication, and absorption as mediators with person—organization fit moderating the mediator → behavior path. The interaction terms were negative and significant (vigor b = −0.102, dedication b = −0.102, absorption b = −0.147, all *p* < 0.001). Johnson-Neyman analysis located thresholds on the mean-centered fit scale at 0.335 (vigor), 0.356 (dedication), and 0.459 (absorption): below each threshold the facet's simple effect on proactive change was significant, above it was not. Consistent with this, the corresponding conditional indirect effects were significant at low fit, weaker at the mean, and non-significant at high fit across all three facets. These facet-level results converge with the global pattern (J-N = 0.433), indicating that vigor, dedication, and absorption are most behaviorally consequential when person-organization fit is lower.

To ensure the rigor of the results, this study also used AMOS software to verify the moderating effect via a Bootstrap test. The parameters were set as follows. The sampling was set to 5,000 times and the confidence level was set at 95%. The main fitting indices of the model are as follows: CMIN/DF = 1.145, RMSEA = 0.019, IFI = 0.991, TLI = 0.990, CFI = 0.991, NFI = 0.932, RFI = 0.926, GFI = 0.922, PNFI = 0.863, and PCFI = 0.918 (CMIN/DF < 3.0, RMSEA < 0.08, IFI > 0.9, TLI > 0.9, CFI > 0.9, NFI > 0.9, RFI > 0.9, GFI > 0.5, PNFI > 0.5, PCFI > 0.5). These results indicate that the model fits well. Detailed results are shown in Table 8. According to Table 8, the standardized coefficients were 0.233 for work engagement, 0.190 for person-organization fit, and −0.205 for the interaction term. The 95% confidence interval for the interaction (−0.371, −0.062) excludes zero (*p* = 0.008), corroborating the hierarchical regression and indicating a robust negative moderation across methods. Together, these findings show a consistent pattern: engagement and fit each relate positively to proactive change on average, yet higher fit dampens the incremental association between engagement and proactive behavior.

**Table 8:** Bootstrap test analysis results.

**Parameter**	**Estimate**	**Lower**	**Upper**	***P***
Work engagement → Proactive change behavior	0.233	0.109	0.370	0.001
Person-organization fit → Proactive change behavior	0.190	0.07	0.298	0.001
Interaction term → Proactive change behavior	−0.205	−0.371	−0.062	0.008

Based on the results from both the hierarchical regression and bootstrap test, it can be concluded that person-organization fit negatively moderates the relationship between work engagement and proactive change behavior. Therefore, hypothesis 5 is supported, indicating that person-organization fit weakens the positive effect of work engagement on proactive change behavior. This should not be interpreted as high-fit employees being less proactive overall. Rather, among high-fit employees, additional engagement yields smaller gains in proactive change than among low-fit employees.

**Figure 3:**
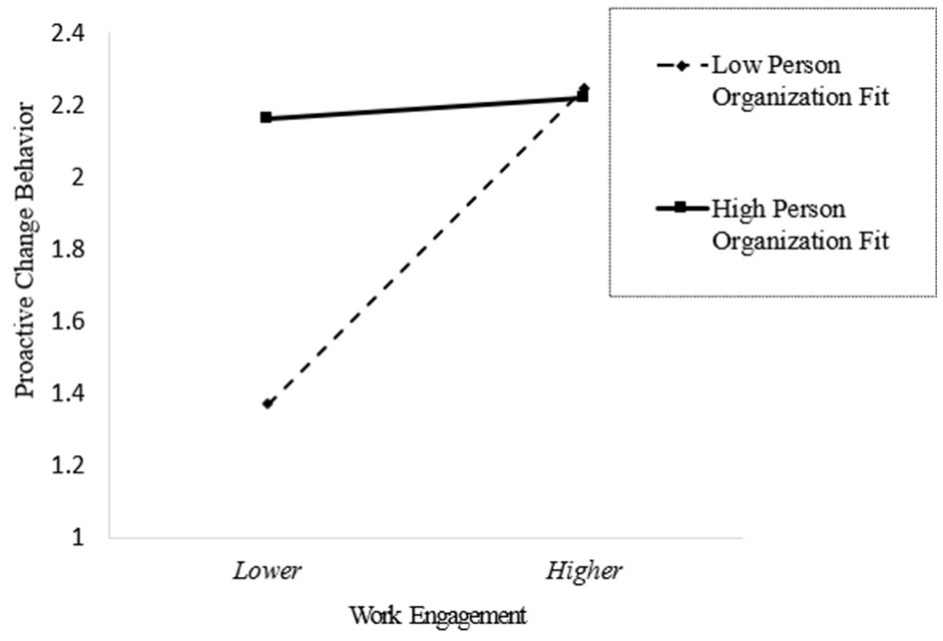
Simple slope analysis of the moderating effect of person-organization fit.

To more clearly illustrate the moderating effect of person-organization fit on employees' work engagement and proactive change behavior, a slope diagram is presented in Figure 3. Figure 3 plots predicted proactive change behavior on the vertical axis against work engagement on the horizontal axis, with separate lines for low (-1 SD) and high (+1 SD) person-organization fit. All covariates are held at their means when generating the predictions. The line corresponding to low fit is visibly steeper, consistent with the significant simple slope within the region identified by the J-N analysis (person-organization fit ≤ 0.433), whereas the line for high fit is comparatively flat, consistent with a non-significant simple slope above that threshold. Thus, person-organization fit plays a negative moderating role in the relationship between work engagement and proactive change behavior. This pattern accords with our framework: at higher fit, clarified norms, satisfied needs, and mature opportunity structures channel engagement toward maintaining effective routines, whereas at lower fit, salient misalignments and unmet needs enable engaged employees to convert effort into change-oriented action.

## 6 Research conclusion and discussion

### 6.1 Analysis of research conclusions

Using AMOS software, this study first analyzed the relationships among change perception, work engagement, and employees' proactive change behavior. The Bootstrap method was employed to test the mediating effect of work engagement on the relationship between change perception and employee's proactive change behavior. Finally, hierarchical regression and the Bootstrap test were used to confirm the moderating effect of person-organization fit on the relationship between work engagement and proactive change behavior. The final test results are summarized in Table 9.

**Table 9:** Hypothesis test results.

**Hypothetical sequence number**	**Research hypothesis**	**Conclusion**
Hypothesis 1	Employees' perception of digital-intelligent HRM change positively influences their proactive change behavior.	Supported
Hypothesis 2	Employees' perception of digital-intelligent HRM change positively influences their work engagement.	Supported
Hypothesis 3	Employees' work engagement significantly influences their proactive change behavior.	Supported
Hypothesis 4	Work engagement mediates the relationship between employees' perception of digital-intelligent HRM change and their proactive change behavior.	Supported
Hypothesis 5	Person-organization fit moderates the relationship between employees' work engagement and proactive change behavior. Specifically, higher levels of fit weaken this positive effect, while lower levels strengthen it.	Supported

Based on the results of hypothesis testing, this study draws the following conclusions. First, employee's perceptions of digital-intelligent HRM change positively influence their proactive change behavior. When employees have a positive perception of organizational change, they are more likely to adopt a positive attitude, enabling them to adapt to new work content, methods, and environments introduced by the change. Second, the perception of digital-intelligent HRM change significantly enhances work engagement. During the change process, if organizations focus on the benefits employees will experience from the change, address their needs, and provide opportunities for growth, they can stimulate positive emotions and encourage employees to invest more effort in their work. Third, employee's work engagement positively affects proactive change behavior. When employees are highly engaged, they demonstrate greater attention and effort in their work. As a result, they are better able to adapt to change and engage in proactive behaviors to support organizational transformation. Fourth, work engagement partially mediates the relationship between change perception and proactive change behavior. Employees who hold positive emotions toward organizational change are more likely to actively participate in their work and become more engaged. Highly engaged employees are more willing to invest time and energy into the organization and are more likely to initiate changes when they identify areas for improvement. Fifth, person-organization fit negatively moderates the positive effect of work engagement on proactive change behavior. A higher degree of matching weakens the positive impact of work engagement on proactive change, whereas a lower degree of matching amplifies this effect.

### 6.2 Managerial implications

First, during digital-intelligent HRM change, leaders should foreground the critical role of employees, especially their active participation in driving change. To understand attitudes and readiness before rollout, conduct a brief baseline that captures change perception and the UWES-9 global score, then repeat the same pulse at key milestones to detect shifts. Use structured methods such as interviews, surveys, and focus groups to surface concerns and expectations. In line with SCT, issue transparent change briefs that specify purpose, scope, success metrics, and timelines, and accompany each major release with a one-page brief and a live Q&A. Maintain continuous communication so that guidance and troubleshooting are timely. For example, when employees report low confidence in new tools, schedule hands-on clinics in the first week and set fixed office hours to resolve early questions quickly.

Second, foster an environment that actively supports digital-intelligent HRM change and make the management system for change efficient and transparent. Publish a detailed roadmap that clarifies objectives, timeline, and expected outcomes, and keep a dedicated intranet page updated with progress and success stories. To translate plans into motivation and engagement consistent with SDT, design practices that support autonomy, competence, and relatedness. Offer teams limited choice over rollout sequencing or configuration to support autonomy. Provide role-specific micro-learning paths, peer coaching, and job aids to build competence. Convene communities of practice or change circles to strengthen relatedness. To mitigate technology overload typical of digital-intelligent HRM change, use staged releases, default notification hygiene, and user-tested interfaces. To address concerns about AI-enabled monitoring, publish a clear data-use notice that specifies what is captured, for what purpose, retention periods, and available appeal channels.

Third, share timely information about the change through channels that employees already use, and convert one-way broadcasting into two-way interaction so that opportunity is created in the AMO sense. Run short stand-up huddles to gather frontline questions, operate a digital suggestion board with weekly review, and form cross-functional improvement squads with authority to trial small process changes for 2 weeks. Recognize specific change-oriented acts such as proposing, testing, and scaling a process improvement, and link this recognition to performance conversations so that motivation converts into observable taking-charge behavior.

Fourth, establish robust communication and feedback mechanisms that connect different organizational levels and tailor interventions by person-organization fit. For lower-fit groups, emphasize sense-making, mentoring, and quick-win pilots that visibly resolve misalignments so that engagement more readily translates into proactive change. For higher-fit groups, prevent routinization and reduced felt urgency by setting explicit improvement OKRs, rotating change-champion roles, and running short innovation sprints so that engagement is channeled into exploration rather than maintenance. Manage execution quality using leading indicators reviewed on a fixed cadence, including change-perception and engagement pulse scores, training completion and practice usage, the number and adoption rate of employee-initiated improvements, and sentiment regarding data-use transparency. When feasible, segment dashboards by fit and team so that managers can direct support where the engagement-to-behavior link is strongest.

### 6.3 Research limitations and future directions

While this study advances understanding of how employee's perceptions of digital-intelligent HRM change relate to work engagement and proactive change behavior, several limitations warrant attention. First, the cross-sectional design allows tests of theoretically specified directional relationships, yet it provides limited leverage on temporal ordering. Future research could employ multi-wave or longitudinal designs, diary studies during staged rollouts of digital-intelligent HRM, or field experiments that manipulate change cues or support to strengthen causal inference.

Second, all focal variables were self-reported, which raises the possibility of common-method bias. Subsequent studies could combine survey data with objective indicators and supervisor or peer ratings of proactive change, introduce temporal separation between predictors and outcomes, and model a latent method factor or include a theoretically unrelated marker variable to diagnose residual bias.

Third, the sample is drawn from a single national context. Cultural features such as collectivism and power distance may shape change perceptions, engagement, and the expression of proactive behaviors. Replications in other countries and multi-country designs would help assess generalizability. Future work should test measurement invariance across cultures and examine whether cultural values moderate key paths in the model. Pairing country-level cultural indices with multi-group SEM would enable formal tests of cross-cultural moderation of the perception → engagement → proactive change pathway.

Fourth, the present analysis focuses on individual-level variables. Organization-level features are likely to shape both perceptions and behavior, including leadership style, change communication climate, high-performance work systems, and the extent of digital-intelligent HRM capability. Multilevel models that incorporate cross-level interactions would clarify how context amplifies or dampens individual mechanisms. Sampling multiple teams or business units per firm would permit random-effects and cross-level moderation tests.

Fifth, work engagement was modeled primarily as a global UWES-9 construct. Although this approach aligns with prior research, the three dimensions, vigor, dedication, and absorption, may differentially predict proactive change behavior. Future research could test facet-level paths, relative weight analyses, bifactor structures, or person-centered profiles to determine whether specific engagement facets are more strongly tied to taking charge under digital change.

Sixth, the moderating effect of person-organization fit was probed with simple slopes and the Johnson-Neyman technique. The Johnson-Neyman analysis in this sample indicated a threshold on the mean-centered fit scale at 0.433, below which the simple effect of engagement on proactive change is statistically significant at the 95 percent level and above which it is not. Because this threshold depends on scaling and the observed range of fit, future studies should report the corresponding value on the raw scale, assess robustness to alternative centering choices, and examine additional boundary conditions such as technology overload, perceived surveillance, or change-oriented leadership. Providing sensitivity checks for different operationalizations of fit would further test stability. For example, categorical terciles vs. continuous centering.

Finally, the digital-intelligent HRM context itself merits deeper operationalization. Future work can include direct measures of technology load, AI-enabled monitoring transparency, and data-use clarity, and test whether these features act as mediators or moderators of the relationships among change perception, engagement, and proactive behavior. Broader sampling frames and probability-based panels would further address potential selection and nonresponse concerns.

## Data Availability

The raw data supporting the conclusions of this article will be made available by the authors, without undue reservation.
